# Murine Gamma-herpesvirus Immortalization of Fetal Liver-Derived B Cells Requires both the Viral Cyclin D Homolog and Latency-Associated Nuclear Antigen

**DOI:** 10.1371/journal.ppat.1002220

**Published:** 2011-09-08

**Authors:** Xiaozhen Liang, Clinton R. Paden, Francine M. Morales, Ryan P. Powers, Joshy Jacob, Samuel H. Speck

**Affiliations:** 1 Emory Vaccine Center, Emory University School of Medicine, Atlanta, Georgia, United States of America; 2 Department of Microbiology & Immunology, Emory University School of Medicine, Atlanta, Georgia, United States of America; 3 Immunology and Molecular Pathogenesis Graduate Program, Emory University School of Medicine, Atlanta, Georgia, United States of America; 4 Microbiology and Molecular Genetics Graduate Program, Emory University School of Medicine, Atlanta, Georgia, United States of America; University of North Carolina at Chapel Hill, United States of America

## Abstract

Human gammaherpesviruses are associated with the development of lymphoproliferative diseases and B cell lymphomas, particularly in immunosuppressed hosts. Understanding the molecular mechanisms by which human gammaherpesviruses cause disease is hampered by the lack of convenient small animal models to study them. However, infection of laboratory strains of mice with the rodent virus murine gammaherpesvirus 68 (MHV68) has been useful in gaining insights into how gammaherpesviruses contribute to the genesis and progression of lymphoproliferative lesions. In this report we make the novel observation that MHV68 infection of murine day 15 fetal liver cells results in their immortalization and differentiation into B plasmablasts that can be propagated indefinitely *in vitro*, and can establish metastasizing lymphomas in mice lacking normal immune competence. The phenotype of the MHV68 immortalized B cell lines is similar to that observed in lymphomas caused by KSHV and resembles the favored phenotype observed during MHV68 infection *in vivo*. All established cell lines maintained the MHV68 genome, with limited viral gene expression and little or no detectable virus production - although virus reactivation could be induced upon crosslinking surface Ig. Notably, transcription of the genes encoding the MHV68 viral cyclin D homolog (v-cyclin) and the homolog of the KSHV latency-associated nuclear antigen (LANA), both of which are conserved among characterized γ2-herpesviruses, could consistently be detected in the established B cell lines. Furthermore, we show that the v-cyclin and LANA homologs are required for MHV68 immortalization of murine B cells. In contrast the M2 gene, which is unique to MHV68 and plays a role in latency and virus reactivation in vivo, was dispensable for B cell immortalization. This new model of gammaherpesvirus-driven B cell immortalization and differentiation in a small animal model establishes an experimental system for detailed investigation of the role of gammaherpesvirus gene products and host responses in the genesis and progression of gammaherpesvirus-associated lymphomas, and presents a convenient system to evaluate therapeutic modalities.

## Author Summary

Herpesviruses are ubiquitous viruses, members of which infect all known mammalian species. A notable feature of all herpesvirus infections is that these infections cannot be cleared and persist for the lifetime of the host. In most cases these infections are benign and often without notable symptoms. However, for a subgroup of herpesviruses – the gammaherpesviruses – some infected individuals develop lymphomas, as well as several other types of cancer. There are two known gammaherpesviruses that infected humans, Epstein-Barr virus (EBV) and Kaposi's sarcoma-associated herpesvirus (KSHV), both of which have been the subject of intensive investigation. However, a major shortcoming of research on these viruses is the absence of an appropriate small animal model since these viruses only infect humans. To circumvent this limitation, infection of mice with a rodent gammaherpesvirus, murine gammaherpesvirus 68 (MHV68), is being characterized. Like EBV and KSHV, MHV68 infection of mice is also associated with the development of lymphoma under some experimental conditions. Here we show for the first time that a hallmark of EBV infection of human B lymphocytes – growth transformation of infected B cells in tissue culture – can be recapitulated by MHV68 infection of murine fetal liver-derived B cells. Furthermore, we identify two MHV68 genes that are required for B cell growth transformation. Finally, we show that MHV68 growth transformed B cell lines cause aggressive lymphomas in mice lacking an intact immune system, but not in immune competent mice. The latter result opens the door for studies on the role of viral genes in driving B cell growth, as well as host immune responses that control outgrowth of MHV68 infected B cells.

## Introduction

The human gammaherpesviruses EBV and KSHV are characterized by their ability to establish latent infections and their close association with a wide variety of malignancies [1]. EBV is the etiologic agent of infectious mononucleosis, and is tightly associated with development of endemic Burkitt's lymphoma, 30–40% of Hodgkin's lymphoma and nearly half of the lymphomas that arise in immunosuppressed patients [2], [3]. KSHV is present in all cases of Kaposi's sarcomas, and is also associated with the development of the rare primary effusion lymphomas (PELs) that occur in some AIDS patients [4], [5]. In addition, KSHV is the etiologic agent of the lymphoproliferative disorder Multicentric Castelman's disease [6]. Both EBV- and KSHV-associated lymphoproliferative diseases and B cell lymphomas mostly occur in immunodeficient individuals, such as arising in HIV infected patients and following organ transplantation. However, because of the narrow tropism of the human gammaherpesviruses, no tractable small animal model is available for dissecting the genesis of EBV- and KSHV-associated lymphomas.

Gammaherpesviruses have been divided into 2 subfamilies – the γ1-herpesviruses (lymphocryptoviruses), of which EBV is a member, and the γ2-herpesviruses (rhadinoviruses), which include KSHV and MHV68. All characterized lymphocryptoviruses share the property of being able to growth transform primary B cells in tissue culture. However, only the T lymphotropic rhadinoviruses (herpesvirus saimiri and herpesvirus ateles) have been shown to transform lymphocyte targets [7], [8]. It has thus been generally assumed that the B lymphotropic rhadinoviruses are non-transforming, and this has significantly impeded studies into the role of KSHV genes involved in the genesis of lymphomas and lymphoproliferative disease. However, in the case of both KSHV and MHV68, the failure to immortalize B cells in culture may be due to their inability to efficiently infect primary B cells in tissue culture [9] and/or the absence of appropriate culture conditions. Here we explore this possibility by targeting virus infection of B cell progenitor populations using fetal liver cells isolated from day 15 embryos cultured in the presence of IL-7 to drive B cell differentiation.

## Results

### Generation of MHV68 immortalized B cell lines

**Figure 1:**
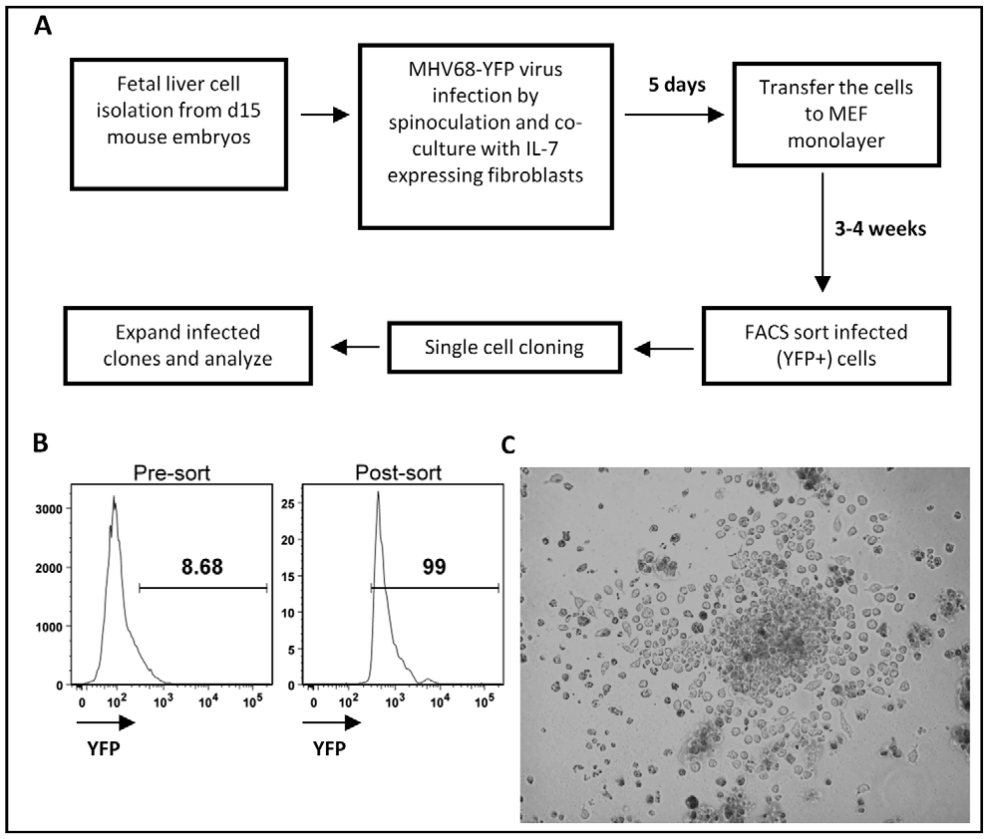
Generation of MHV68 immortalized fetal liver-derived B cell lines. (A) Two stages in vitro culture system for establishing MHV68 infected fetal liver-derived B cell lines. (B) Purification of MHV68-YFP infected fetal liver cells by flow cytometry. Shown are representative pre- and post-sort analyses of YFP-expression. (C) Morphology of a representative MHV68-YFP infected culture at 2.5 weeks post-infection, showing extensive clumping of cells and lymphoblastoid phenotype.

Fetal liver is the major site of B lymphopoiesis during embryonic development, prior to the initiation of B cell development in the bone marrow [10]. The early progenitor cells from murine fetal liver can differentiate into immature B cells upon co-culture with IL-7 producing stromal cells *in vitro* [11], [12] or into mature B cells upon transfer into severe combined immunodeficient (SCID) mice [13], [14]. Using a recombinant MHV68 harboring a YFP expression cassette, pilot experiments demonstrated that MHV68 can infect fetal liver cultures derived from day 15 mouse embryos (data not shown). This led to the development of a two-stage culture strategy in which MHV68 infected fetal liver cells were initially cultured on IL-7 expressing T220 fibroblasts [15] for 4 to 5 days to drive B lineage development, followed by transfer of the infected fetal liver cells onto mouse embryo fibroblasts (MEFs) (Fig. 1A). The T220 fibroblast and MEF monolayers are permissive for MHV68 replication, and thus were destroyed after several days of co-culture with the infected fetal liver cells. The infected fetal liver cells were maintained as a bulk culture for 3-4 weeks to generate a sufficient population of infected YFP+ cells that could then purified by flow cytometry (Fig. 1B). Cell lines were generated from sorted YFP+ cells by either single cell sorting or limiting dilution cell culture. Cell lines derived by this approach displayed lymphoblastoid morphology with ovoid or slightly elongated shape and the presence of villipodia projecting from the cell surface (Fig. 1C), similar to the appearance of EBV immortalized lymphoblastoid cell lines [16].

**Figure 2:**
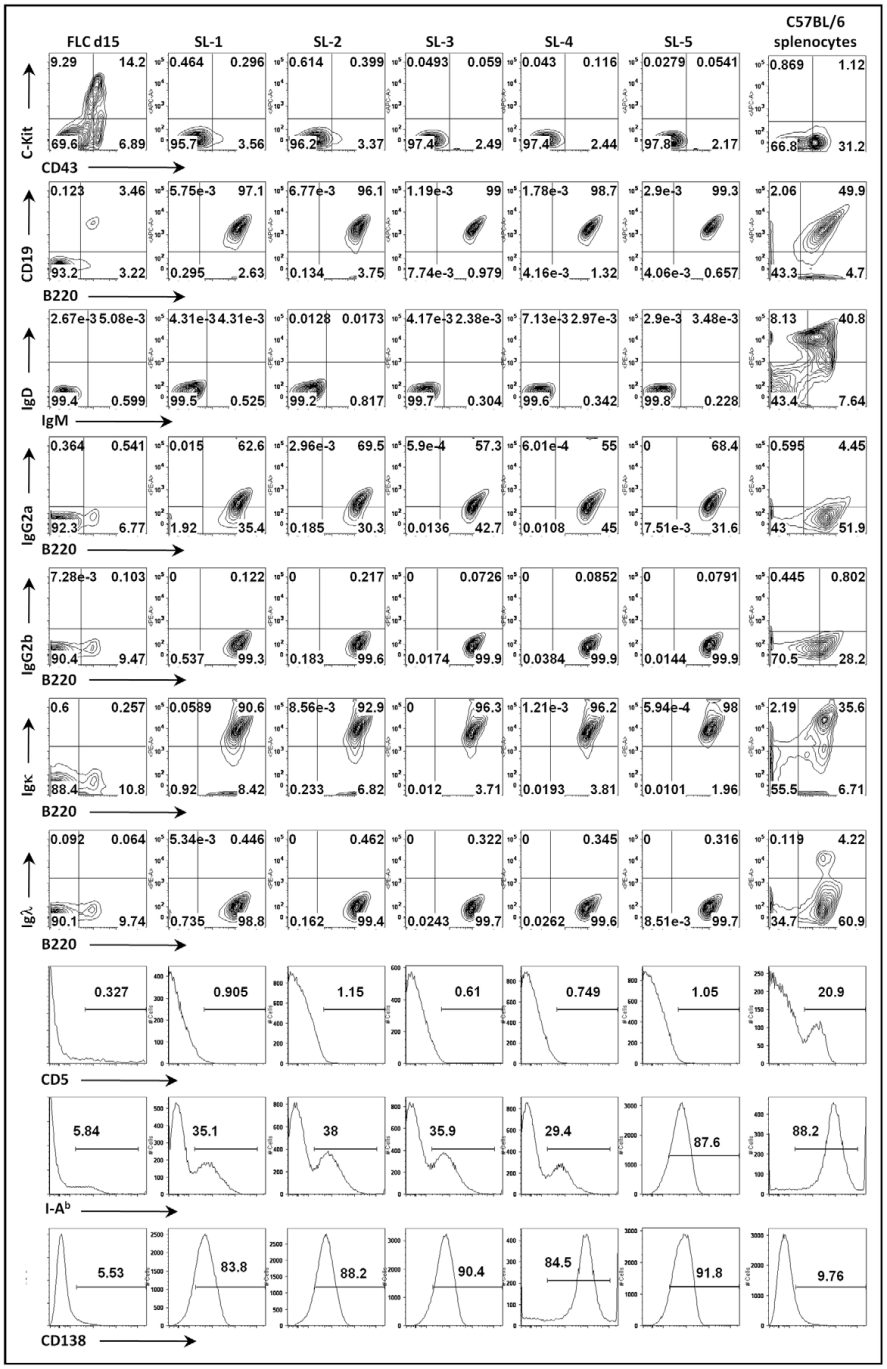
Surface phenotyping MHV68 infected cell lines derived from infection of day 15 fetal liver cells. Cell surface expression was analyzed for individual cell lines derived from single cell cloning by flow cytometry. Day 15 fetal liver cells (FLC d15) and splenocytes harvested from naive C57BL/6 mice (BL6 splenocytes) were used as staining controls.

Surface staining demonstrated that all the cell lines established bore cell surface markers characteristic of B lymphocytes (B220+/CD19+/c-kit-/CD43-). Surprisingly, all cell lines were sIgD-/sIgM-, suggesting that they either were arrested at an early stage of development or had undergone isotype switching (Fig. 2 and data not shown). Subsequent staining with a panel of isotype specific antibodies revealed that these cell lines expressed surface immunoglobulin, having undergone class switch recombination to IgG2a (Fig. 2 and data not shown). Staining for light chain usage revealed that all the cell lines were Igκ+ and Igλ- (Fig. 2). Notably, even though these cell lines arose from fetal liver, all were CD5- (Fig. 2). In addition, they were I-A^b^ intermediate or low and CD138 (syndecan) intermediate or high (Fig. 2), suggesting that these cell lines have differentiated toward a plasmablast phenotype. Consistent with this interpretation, ELISA analyses of the culture supernatants demonstrated significant levels of secreted IgG2a and kappa light chain (Fig. S1). Furthermore, cytoplamic expression of IgG2a was detected in MHV68 immortalized cell lines by immunofluorescent staining (Fig. S2). We also examined the expression of germline transcripts directed by the I promoter of each isotype (Fig. S3A), which revealed the presence of IgG2a germline transcripts and low levels of IgG3 germ line transcripts (Fig. S3A). After immunoglobulin class switch recombination (CSR), post-switch transcripts containing the Iµ exon spliced to the 5′ exon of the relevant rearranged isotype (directed by constitutive active Iµ promoter) are generated [17], [18]. Correspondingly, we readily detected the expression of IgG2a post-switch transcripts (Fig. S3A), and not any of the other immunoglobulin isotypes (data not shown). The IgG2a post-switch transcripts were further confirmed by cloning and sequencing the amplified PCR product (Fig. S3B). Finally, consistent with the observation of class switch recombination, RT-PCR analyses detected the expression of AID in these cell lines (data not shown). Taken together, these data support the conclusion that MHV68 infection of a fetal liver B cell progenitor population(s) not only leads to their immortalization, but also directs these cells to differentiate into IgG2a-expressing plasmablasts.

Next, we determined the clonality of B cell lines transformed by MHV68. Transformation of B lymphocytes by murine retroviruses leads to outgrowth of transformed lines that are oligo or pauciclonal in nature [19]. To determine the clonality of the B cell clones we used primer sets capable of amplifying >98% of rearranged Ig gene segments. We amplified and sequenced Ig H chain gene segments from 2 of the established MHV68-transformed B cell lines (Fig. S4). Surprisingly, we found that these B cell lines exhibited high clonal diversity. Of the 16 H chain gene segment sequences obtained, junction analyses indicated that there were 10 unique junctions (Table S1). Further analysis of J_H_ usage, as well as V-region nucleotide sequence comparison, revealed an additional 2 sequences which were determined to be unique. Thus, 12 out of 16 (75%) heavy chain sequences were unique. This data suggests that, unlike murine leukemia virus, MHV68 transformation yields a polyclonal population of immortalized B cells. This observed polyclonality could be due to one of the two following reasons: (a) MHV68 targets and immortalizes a variety of mature B cells; or (b) the virus immortalizes a progenitor cell, prior to VDJ rearrangement. The latter seems likely since multiple junction sequences were obtained within each of the 2 cloned cell lines analyzed, although it is possible that only those wells that were seeded with more than one MHV68 infected B cell grew out as cell lines. We also analyzed V_H_ segment usage from transformed B cells and all of them were confined to the VH5 family (Table S2). This skewing most likely reflects the predominant rearrangement of the D-proximal VH5 family V gene segments in fetal liver, rather than MHV68 transformation being restricted to select BCR-bearing B cells.

### Retention of viral genome and ability to trigger virus reactivation in MHV68 immortalized B cell lines

**Figure 3:**
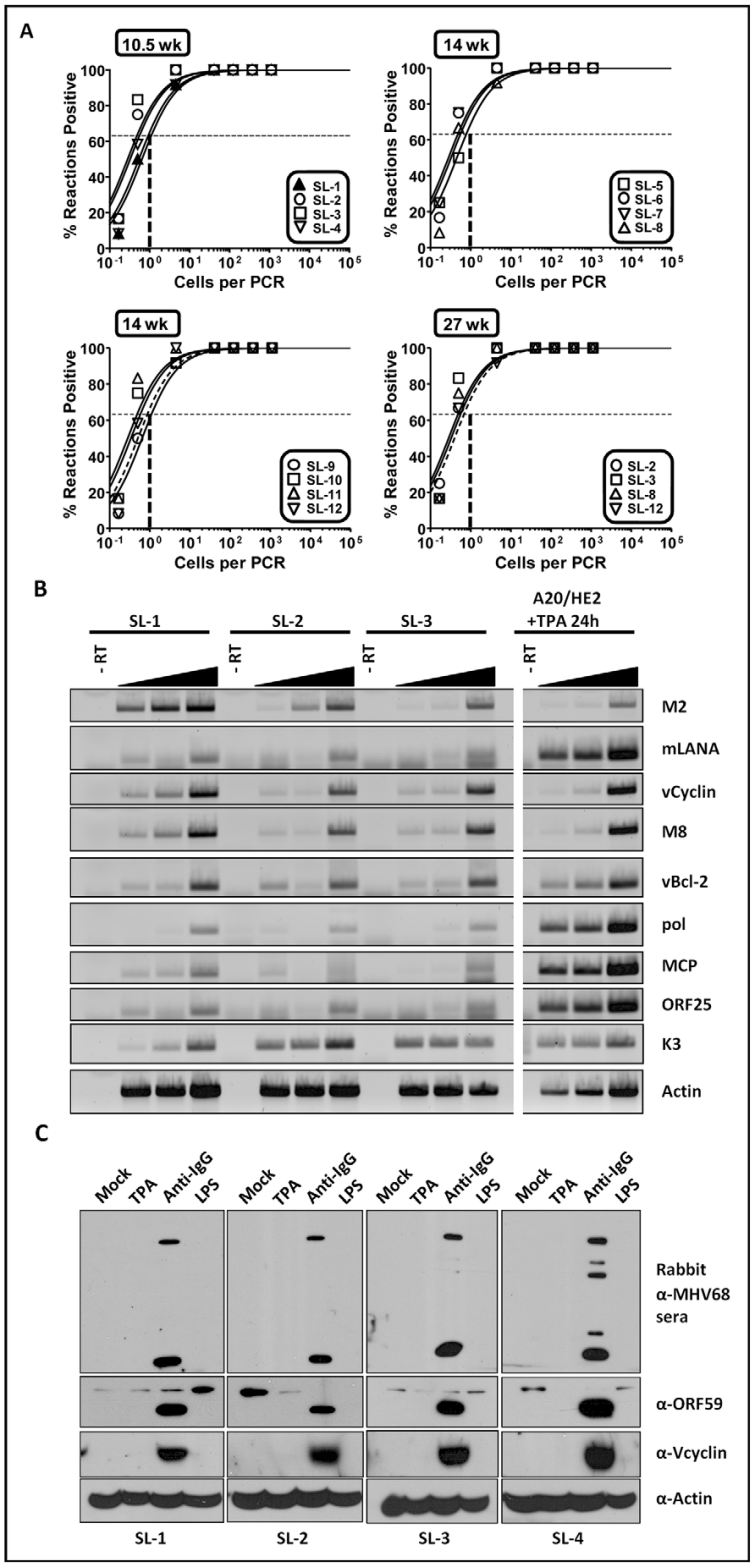
Analyses of viral genome frequency, viral gene expression and induction of virus reactivation from MHV68 immortalized B cell lines. (A) The MHV68 genome is stably maintained in the established fetal-liver derived B cell lines. Shown was limiting dilution PCR detection of the MHV68 genome. The intersection of the horizontal and vertical dashed lines depicts the predicted point, based on a Poisson distribution, where every cell harbors viral genome. (B) Semi-quantitative RT-PCR of a panel of viral genes known to be associated with either virus replication and/or latency. cDNA synthesized from total RNA isolated from individual cell lines was serially diluted 1∶1, 1∶5, or 1∶25 prior to PCR amplification. (C) Immunoblot analyses of viral lytic gene expression after treating cells with candidate reactivation stimuli. The cells were stimulated with the indicated reagents for 72 hr, followed by immunoblot detection with either a rabbit anti-MHV68 antiserum, a chicken anti-ORF59 antibody, a rabbit anti-vCyclin antibody or a mouse anti-actin antibody.

Since mock infected day 15 fetal liver cell cultures did not survive and expand following transfer of the cultures to mouse embryo fibroblast feeder layers, we conclude that virus infection is required for outgrowth of fetal liver-derived B cell lines using the assay described above (Fig. 1A). To determine whether there is an ongoing requirement for virus infection, we assessed the frequency of cells harboring viral genome using a limiting-dilution PCR analysis (Fig. 3A). These analyses demonstrated that the viral genome is faithfully maintained in all the cell lines generated, even after serial passage for more than 6 months in tissue culture (Fig. 3A). To assess viral gene expression in these B cell lines, semi-quantitative RT-PCR analyses were performed for a panel of candidate latency-associated and known replication-associated viral genes (Fig. 3B). Tetradecanoyl-phorbol-13-acetate (TPA)-treated A20-HE cells [20] (generated by MHV68 infection of the murine A20 B cell line) served as a positive control for expression of replication cycle-associated viral transcripts in the setting of virus reactivation from B cells (Fig. 3B). Expression of the replication-associated genes encoding the viral DNA polymerase (pol), major capsid protein (MCP) and ORF25 were only detected at low levels (Fig. 3B) in the MHV68 transformed B cell lines, consistent with the majority of the infected cells being latently infected. This was further underscored by detection of transcripts for the known latency-associated genes M2, ORF 73 encoding the latency-associated nuclear antigen (mLANA), and the viral bcl-2 homolog (M11) (Fig. 3B). In addition, expression of viral genes that may play a role in both latency and virus replication (v-cyclin, M8 and K3) were also detected at significant levels in most of the transformed cell lines.

A hallmark of herpesvirus latency is the ability to enter the viral replication program (reactivation) given the appropriate stimulus [21]. We assessed a panel of MHV68 transformed B cells lines for induction of lytic antigen expression following treatment with either TPA, crosslinking surface immunoglobulin, or addition of lipopolysaccharide (LPS) to the culture medium. Somewhat surprisingly, TPA did not trigger virus reactivation (Fig. 3C). However, anti-Ig treatment was able to induce virus reactivation in all cell lines tested - although output virus titers were low (Fig. 3C and data not shown). This is reminiscent of the difficulty in triggering EBV reactivation from LCLs generated using umbilical cord lymphocytes compared to those generated from adult peripheral B cells [22].

### Viral cyclin and mLANA are required for MHV68 immortalization of murine day 15 fetal liver-derived B cells

**Figure 4:**
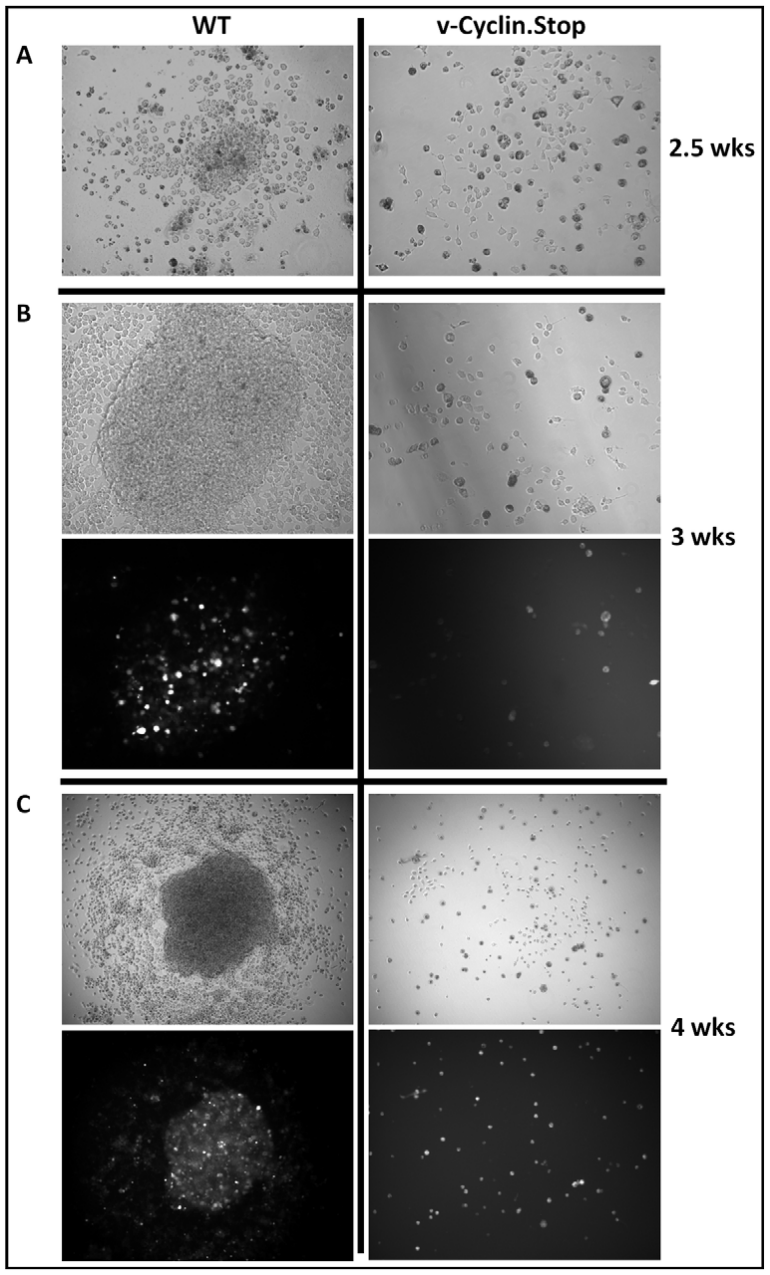
The MHV68 cyclin D homolog is required for immortalization of fetal liver-derived B cells. Fetal liver (FL) cells, isolated from embryos at day 15 of gestation, were infected with wild-type (WT) MHV68-YFP or v-Cyclin.Stop-YFP viruses at an MOI of 10 by spinoculation. (A) Colonies were readily apparent in WT infected FL cell cultures by 2.5 weeks post-infection, but not in v-Cyclin.Stop-infected FL cell cultures. (B and C) The colonies derived from WT-infected FL cells grew rapidly and contained numerous YFP bright cells. In contrast, there was little evidence of cellular proliferation or colony formation in the v-Cyclin.Stop infected FL cell cultures. The images were taken at magnification 200X (A) or 100X (B and C), and both brightfield (top panels) and YFP-fluorescence images (bottom panels) are shown.

To begin to assess the viral requirements for B cell immortalization, we considered candidate latency-associated viral genes for which we consistently detected transcripts in the MHV68 immortalized B cell lines (Fig. 3) and whose functions would contribute to B cell immortalization. As such, we chose three viral genes to analyze - two genes that are conserved among all known rhadinoviruses (v-cyclin and LANA) and one gene that is unique to Old World rodent rhadinoviruses (M2). The v-cyclin gene is an obvious candidate to examine since we have previously shown that it is capable of inducing lymphomas when expression was targeted to T cells in transgenic mice [23]. Similarly, mLANA would be expected to play a central role in B cell immortalization based on the role of KSHV LANA in maintenance of the viral episome during latency, as well as its role in regulating lytic gene expression. The other candidate gene we examined is the M2 gene, a novel MHV68 gene product that we have shown can enhance survival and proliferation of primary murine B cells [24]. Day 15 fetal liver cells were infected with either wild type virus, the v-cyclin null virus (v-cyclin,Stop), mLANA null virus (mLANA.Stop) or the M2 null virus (M2.Stop), and cultured as described above. Notably, while wild type virus infected B cells formed many colonies which were easily detected by light microscopy, the v-cyclin.Stop virus infected B cells formed a small number of disorganized colonies that were sparse in cell number and failed to expand (Fig. 4 and Table 1). Fetal liver-derived B cells infected with the mLANA.Stop virus also failed to generate any infected foci, and YFP+ cells were rapidly lost in these cultures (Table 1). In contrast, M2.Stop virus infected fetal liver-derived B cells formed colonies from which YFP+ cells were isolated and immortalized cell lines established (Table 1 and data not shown). It is perhaps notable that the generation of immortalized cell lines from the M2.Stop cultures appeared less efficient than that observed with wild type MHV68 – however, this interpretation should be taken with some caution because the assay used was not designed to be quantitative.

**Table 1:** Immortalization of FL-derived B cells.

Virus	Colony formation
WT	+++
M2.Stop	++
v-cyclin.Stop	+/-
mLANA.Stop	-

### Induction of lymphomas upon adoptive transfer of MHV68 immortalized B cell lines into T cell-deficient mice

**Figure 5:**
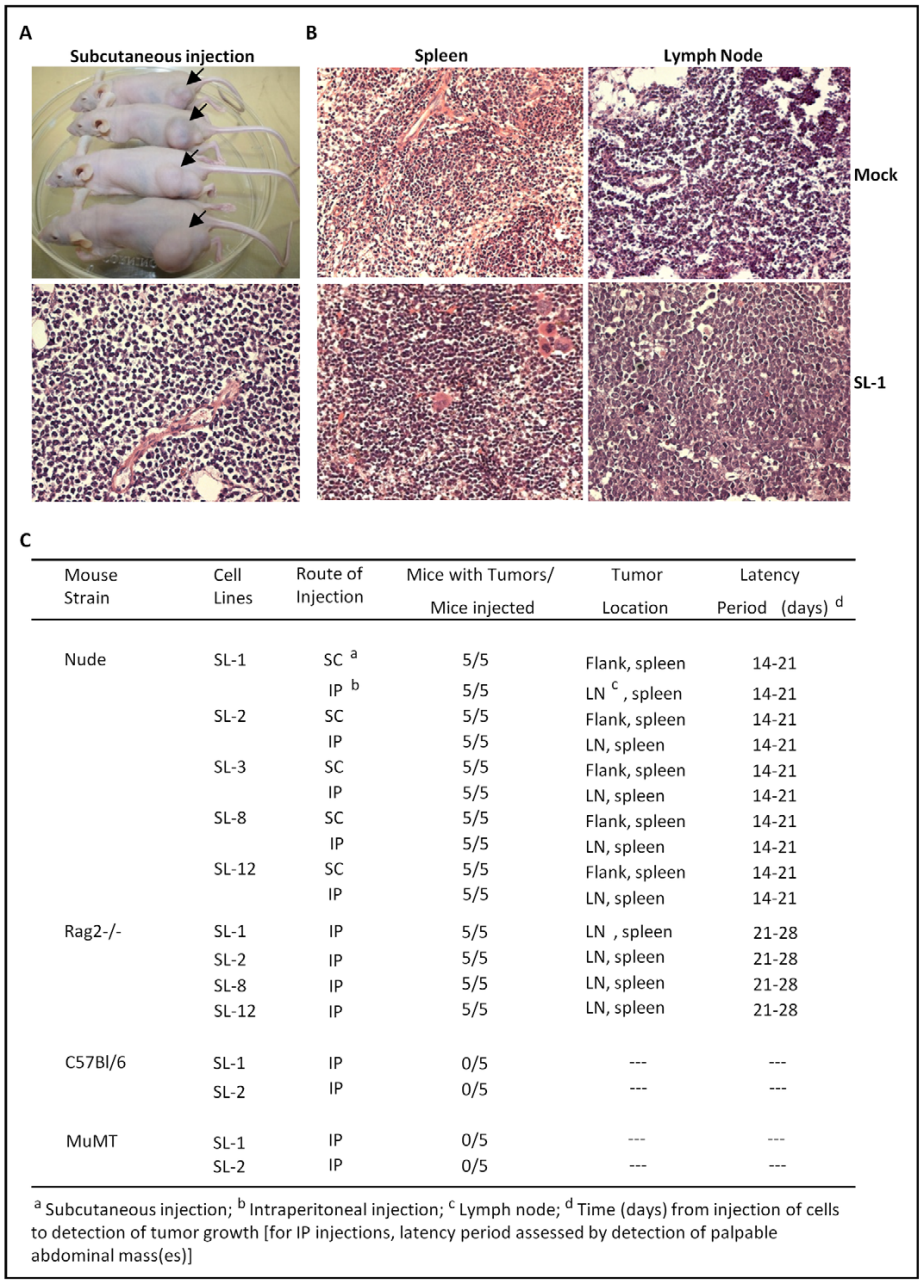
Induction of lymphomas by MHV68 immortalized FL cell lines in athymic nude and Rag 2-deficient mice. (A) Subcutaneous tumors at the site of injection on the rear flank of Nu/J mice (upper panel). Also shown in a representative H&E stained tumor section (lower panel). (B) Representative H&E stained tumor sections of either mock treated or Nu/J mice inoculated intraperitoneally with the SL-1 MHV68 transformed B cell line. Lymphomas were apparent in both the spleen and lymph node in Nu/J mice injected with MHV68 immortalized FL cell lines. (C) Summary of lymphoma induction using the indiciated MHV68 immortalized B cell lines inoculated into athymic nude, Rag 2-deficient, C57Bl/6 or MuMT (B cell-deficient) mice.

During EBV infection in immunocompetent individuals, outgrowth of EBV growth transformed B cells is tightly controlled by a vigorous CD8+ cytotoxic T lymphocyte (CTL) response which targets viral antigens expressed in these B cells. Immune suppression leading to diminished EBV-specific CTL responses thus appears to be central in the progression of EBV-associated lymphoproliferative disease and the genesis of lymphomas in AIDS patients and organ transplant recipients. To determine whether the MHV68 growth transformed B cell lines recapitulate features of EBV in immuncompromised individuals, we assessed the ability of the cloned cell lines to form tumors when injected either subcutaneously or intraperitoneally into immunocompromised mice (athymic nude mice or RAG2-/- mice) (Fig. 5). Importantly, as expected, no tumors were observed when MHV68 infected fetal liver-derived B cell lines were injected into either B cell-deficient mice (µMT) or wild-type C57BL/6 mice (Fig. 5C) – demonstrating that an intact host immune system is able to control outgrowth of MHV68 transformed B cell lines. This is consistent with the induction of T cell-mediated control of gammaherpesvirus transformed B cells, as observed following EBV infection of immunocompetent individuals. However, as expected, all of the MHV68 infected B cell lines tested were tumorigenic in both athymic nude (T cell-deficient) and RAG2-deficient (B and T cell-deficient) mice, with the appearance of subcutaneous tumors or tumors in lymph nodes appearing 14 to 28 days after injection of cells (Fig. 5). All tumors showed characteristics of lymphomas histopathologically, and all inoculated mice (following either subcutaneous or intraperitoneal injection of tumor cells) showed metastasis of these lymphomas to the spleen (Fig. 5B). The presence of B cells in these lesions was verified by immunohistochemical staining of tumor tissue sections. All tumor tissue sections were homogeneously infiltrated with B220-expressing B cells (Fig. S5). A low level of CD8+ T cells were apparent in the spleens of mock infected nude mice, but not in spleens recovered from mice inoculated with the MHV68-infected fetal liver-derived B cell lines (Fig. S5). Explanted tumors could readily be cultured *ex vivo*, were viral genome positive, and exhibited a similar pattern of viral gene and cell surface marker to the parental cloned cell lines (Fig. S6 and data not shown). However, it is notable that all the explanted tumor cell lines had upregulated expression of CD5 and expressed higher levels of MHC II than the parent cell lines (Fig. S6B). The upregulation in CD5 expression resonates with the ability of fetal liver-derived B cell progenitors to effectively reconstitute CD5^+^ B cells *in vivo* [25], [26].

## Discussion

The ability to study the pathogenesis of EBV and KSHV in the infected host has been significantly hampered by the lack of robust small animal models. Although significant progress has been made in the generation of humanized mice, most notably using NOD/SCID/IF2Rγ^null^ mice as recipients of human hematopoietic stem cells, the T cell responses that are generated are clearly not normal and, at least in the case of EBV infection, are ultimately insufficient to control virus infection [27], [28], [29]. Similarly, while a number of important insights have been gained from the generation of various transgenic mice (e.g., those harboring the KSHV LANA gene and associated regulatory elements) [30], [31], [32], [33], [34], this approach cannot assess the contribution that host immune control and selection play in the genesis of gammaherpesvirus-associated lymphoproliferative diseases. Non-human primate models offer the possibility to fully explore the pathogenesis of gammaherpesviruses that are very closely related to the human pathogens (rhesus lymphocryptovirus and rhesus rhadinovirus), but to date only limited studies have been carried out using these models - presumably due to the substantial expense associated with breeding gammaherpesvirus-free colonies of Rhesus macaques. Thus, the MHV68-mouse model stands alone as the only tractable small animal model that has been extensively studied.

A detailed analysis of viral gene expression in established MHV68 immortalized B cell lines will be required before all the viral genes required for immortalization can be determined. However, our initial characterization of viral transcripts detected in these B cell lines identified several viral genes whose products are candidates for being involved in driving B cell survival and/or growth (v-cyclin, mLANA, vBcl-2 and M2). Furthermore, we have shown here that both the v-cyclin and mLANA are required for immortalization of fetal liver-derived B cells. The latency genes K-cyclin and LANA, homologs of MHV68 v-cyclin and mLANA, are expressed in all KSHV-associated maglinancies, Kaposi's sarcomas (KS), primary effusion lymphomas (PEL) and multicentric Castleman's diseases (MCD) [35], [36], [37], [38]. Furthermore K-cyclin, which modulates the cell-cycle by phosphorylation of p27 in PEL cells, has been implicated in the development of KS tumors and the induction of lymphomas by cooperating with p53 loss [39], [40], [41]. The MHV68 v-cyclin has also been shown to be an oncogene [23]; mice harboring a v-cyclin transgene under the control of the lck proximal promoter (active during early T cell development) develop high grade lymphoblastic lymphomas. Here we detected the expression of v-cyclin mRNA prior to the induction of reactivation and expression of v-cyclin protein after anti-Ig cross-linking to induce reactivation in MHV68 transformed FL cells. Furthermore, v-cyclin null virus induced abortive transformation of fetal liver-derived B cells, indicating that v-cyclin is essential for MHV68 growth transformation of murine B cells. In this light, the v-cyclin gene has been shown to promote MHV68-infected primary endothelial cell survival [42]. The mechanism(s) by which v-cyclin contributes to B cell immortalization is currently under investigation, but likely involves a cdk-dependent activity.

LANA, a conserved rhadinovirus gene, has been shown to be expressed in every KSHV-associated malignancy examined. Thus, LANA has a strong likelihood of being critically involved in the formation of rhadinovirus tumors, but to date, this fact has not been shown in the context of virus infection. Our data demonstrate a requirement for mLANA in MHV68 growth transformation of murine B cells, suggesting the conserved function to KSHV LANA homolog. KSHV LANA exhibits transforming properties in primary rat fibroblasts [43]. Similar to v-cyclin-expressing transgenic mice, the KSHV LANA homolog has been shown to be a potent inducer of B cell tumors when expressed in transgenic mice [35]. Further, LANA was shown to give a significant survival advantage to B cells responding to antigen [44]. The mechanisms responsible have not yet been clearly identified, but LANA has been observed interacting with many proteins involved in cell growth pathways, inhibiting p53-mediated apoptosis [45] and regulating the cell cycle [43], [46], [47]. During primary infection of fibroblasts, mLANA has been shown to be required for efficient replication of the virus, and in the absence of mLANA, the virus shows dysregulated expression of viral genes and an early hyperlytic phenotype, resulting in a lower output of virus overall [48]. These data are consistent with the observation in KSHV that LANA may interact directly with and downregulate transcription from the immediate-early ORF50 promoter [49]. These data, taken together argue strongly for the requirement of mLANA in the efficient development of MHV68-derived tumors. mLANA likely sets the stage for transformation, by interacting with and altering pro-growth and anti-apoptotic pathways, stabilizing the cell and by turning off lytic genes, preventing the infected FL cells from succumbing to lytic infection. This is not to say LANA is a strict requirement for rhadinovirus transformation--MHV68 can establish latency in the absence of mLANA [50] and EBV can establish LCLs in the absence of the major latency-associated protein EBNA-1, though the process is less efficient by several thousandfold [51]. Further investigation of the role of mLANA in MHV68 transformation is planned.

Gammaherpesviruses encode homologs of cellular antiapoptotic BCL-2 proteins (vBCL-2), inhibiting apoptosis in response to diverse stimuli. EBV-encoded BHRF1 and BALF1, two vBCL-2 homologs, KSHV-encoded vBCL2, as well as MHV68-encoded vBCL-2 exhibit antiapoptotic functions which protect the infected cells from apoptosis induced by the host anti-viral immune responses [52]. MHV68 vBCL-2 has been shown to be important during MHV68 chronic infection and disease, but not acute infection [53]. Recently, it has also been shown to antagonize the host autophagy during MHV68 persistent infection [54]. The oncogenic potential of vBCL-2 is an obvious candidate for analysis in B cell immortalization.

There are also strong parallels between the observed phenotype of the MHV68 transformed B cell lines and the previously characterized impact of M2 protein expression on B cell growth and differentiation [24], [55]. We have previously shown that expression of M2 in primary murine B cells enhances survival and proliferation, both of which are dependent on M2 induction of cellular IL-10 expression [24]. M2 expression also drives B cell differentiation – including CSR, downregulation of MHC II and B220 surface expression and upregulation in surface syndecan expression and secreted IgG, consistent with differentiation toward a plasmablast phenotype [24]. Furthermore, the observation that the 12 cell lines characterized had isotype switched to IgG2a recapitulates a bias observed during MHV68 infection *in vivo*, where nearly half of the infected B cells detected in the spleen at the peak of latency are IgG2a+. Importantly, this phenotype in vivo is dependent on a functional M2 gene product. Finally, we have previously shown that the presence of MHV68 in plasma cells during virus infection in mice is dependent on the presence in the virus of a functional M2 gene [55]. Studies are underway to define how viral genes regulate MHV68 immortalization of fetal liver-derived B cells.

In summary, we have demonstrated that infection of fetal liver progenitor B cells overcomes a barrier previously encountered in studies attempting to generate MHV68 transformed B cell lines using mature B lymphocytes [9]. This may have implications to studies of KSHV, which to date have failed to show any B cell transforming activity. The ability to transform murine B cells with MHV68 opens the door to developing the mouse model of gammaherpesvirus infection to dissect the mechanisms through which gammaherpesviruses manipulate B cell biology and contribute to B cell lymphomagenesis. Importantly, the ability of these cell lines to generate tumors in athymic nude mice and Rag2-/- mice, but not in either C57Bl/6 or B cell-deficient mice, underscores the critical role of T cells in controlling the outgrowth of MHV68 latently infected B cells. The adoptive transfer of MHV68 immortalized B cell lines has the added advantage that there appears to be little or no reactivation disease apparent in these animals – an issue that complicates studies of MHV68 infected immunocompromised mice where a number of cellular reservoirs (e.g., infected macrophages) ultimately contribute significantly in the setting of immunocompromise to disease associated with MHV68 replication [56]. The latter likely obscures, at least in some settings, the detection and/or development of MHV68-associated lymphomas since this may lag behind the development of end-stage reactivation disease. Finally, we anticipate that this new model will also facilitate studies to identify cellular factors contributing to the generation of gammaherpesvirus-associated B cell lymphomas.

## Materials and Methods

### Ethics statement

This study was carried out in strict accordance with the recommendations in the Guide for the Care and Use of Laboratory Animals of the National Institutes of Health. The protocol was approved by the Emory University Institutional Animal Care and Use Committee, and in accordance with established guidelines and policies at Emory University School of Medicine (Protocol Number: 046-2010).

### Viruses, cell culture, mice

Murine gammaherpesvirus 68-YFP (MHV68-YFP), M2.Stop-YFP, mLANA.Stop-YFP, and v-Cyclin.Stop-YFP viruses were prepared as previously described [55]. C57Bl/6 mice, Nu/J nude and RAG-2-/- mice were sterile housed, treated and bred with the approval of the Emory University Institutional Animal Care and Use Committee, and in accordance with established guidelines and policies at Emory University School of Medicine (Atlanta, GA). IL-7-expressing T220 fibroblasts [15] and mouse embryo fibroblasts (MEF) were cultured in DMEM medium with 10% FCS, 100 U/ml penicillin and 100 µg/ml streptomycin.

### Generation of MHV68 immortalized fetal liver cell lines

Fetal liver (FL) cells were obtained at day 15 of gestation. The dispersed cells were washed with PBS and resuspended in RPMI 1640 medium with 5% FBS, 100 U/ml penicillin and100 µg/ml streptomycin. Fetal liver cells were infected at an MOI = 10 with either wild type or specific MHV68 mutants. All viruses used harbored a yellow fluorescent protein transgene expression cassette introduced between orf 29a and orf 27 in the viral genome, as previously described [57]. Fetal liver cells were mixed with virus in the presence of polybrene (5 µg/ml), and plated into 6-well plates seeded with IL-7 expressing fibroblasts (either the T220 or LTK cell lines). Spinoculation was carried out by centrifugation at 1800rpm for 1 hr at room temperature, followed by incubation at 37°C for 5 days. At day 5 post-infection, live cells were transferred onto MEF monolayers for 3 to 4 days, followed by culturing for 2 to 4 weeks to obtain sufficient cell numbers to allow MHV68 infected YFP-positive cells to be recovered by flow cytometry. During this expansion the cultures were fed weekly. The sorted YFP positive cells were subcloned in 96-well plates, either by limiting dilution cloning or single cell sorting by flow cytometry. Cell lines were recovered and further cultured in RPMI 1640 medium with 5% FBS, 100 U/ml penicillin and 100 µg/ml streptomycin.

### Flow cytometry and fluorescence activated cell sorting (FACS)

Flow cytometric analyses were done as previously described [55]. Briefly, single-cell suspensions were incubated with PE-, PerCP-, APC, APC-Cy7, and/or PacBlue-conjugated mAb on ice for 15 min, and then washed with 0.5% FBS/PBS. The stained cells were subjected to analyses on a LSRII flow cytometer (BD Biosciences). The antibodies used for staining were purchased from BD Biosciences except where noted, including: APC-anti-CD117 (C-kit) , PE-anti-CD43, APC-anti-CD19, APC-Cy7-anti- B220, PerCP-anti-IgM, PE-anti-IgD, PerCP-anti-CD5, PE-anti-IA^b^, PE-anit-CD138, PE-anti-IgG2a (Southern Biotech), PE-anti-IgG2b (Southern Biotech), PE-anti-Igκ (Southern Biotech), anti-Igλ (Southern Biotech). For YFP-positive cell sorting, the single cell suspensions were resuspended with 0.5%FBS/PBS and directly subjected to separation on a FACSAria™ II flow cytometer (BD Biosciences).

### Analysis of immunoglobulin gene rearrangements

RNA was prepared from two representative MHV68 transformed B cell lines, and RT-PCR performed to amplify VH gene segments. Primers for immunoglobulin heavy chain variable regions were made by creating consensus primers from VH sequences obtained from the international ImMunoGeneTics Information system (www.imgt.org). Consensus primers were designed for each VH family and specific primers were made for all four Jh segments. PCR amplification for heavy chains were run using pooled VH and JH primers and high-fidelity Taq polymerase. PCR products were cloned and sequenced. Heavy chain sequences were input into the IMGT program V-QUEST for analysis.

### Immunoglobulin Isotyping

1-2×10^6^ cells of representative MHV68 immortalized FL-derived cell lines were plated in 6-well plates, supernatants were collected after culturing cells for 5 days and subjected to antibody isotyping using an ELISA mouse mAb isotyping kit (Thermo scientific), based on manufacturer's instructions. Each cell line was analyzed in triplicate.

### Immunofluorescence staining, immunohistopathology and immunohistochemistry

For histopathological analyses, tissues were fixed in formalin, dehydrated, and embedded in paraffin. The embedded tissues were cut at about 5 µm of thickness prior to staining with hematoxylin and eosin for histopathological analyses. For immunohistochemical analyses, the sections were deparaffinized, rehydrated and incubated with citrate buffer (pH 6) for antigen retrieval before the sections were subjected to immunofluorescence staining. The stained slides were dehydrated in 95%, 100% ethanol and xylene. Anti-fade mounting medium (Invitrogen) with DAPI counterstaining (Invitrogen, USA) were used prior to examining tissue sections on a LSM510 META confocal microscope (Zeiss). The reagents used for the tissue section staining included: biotin anti-mouse CD8α (BD Biosciences), purified rat anti-mouse CD45R (B220) (BD Biosciences), Alexa fluor 647-conjugated streptavidin and Alexa fluor 555-conjugated secondary antibodies (Invitrogen).

### Limiting dilution PCR analyses

The frequency of MHV68 genome-positive cells was determined using a previously described nested PCR assay (LD-PCR) [58]. Briefly, cells were counted, resuspended in an isotonic solution, and diluted into a background of 10^4^ uninfected NIH 3T12 cells. Following cell lysis with proteinase K, two rounds of nested PCR were performed on each sample to detect the presence of the MHV68 ORF50. To ensure sufficient sensitivity of the nested PCR reaction, 10, 1, or 0.1 copies of a gene 50 containing plasmid (p*Bam*H I N) were diluted into a background of 10^4^ uninfected cells and analyzed in parallel with the experimental sample.

### RT-PCR and semi-quantitative RT-PCR

Total RNA was extracted from the cells using TRIzol reagent (Invitrogen) according to manufacturer's instructions. 2 µg RNA were used for first-strand cDNA synthesis (Invitrogen) prior to PCR analysis. For semi-quantitative RT-PCR analyses of viral gene expression, viral transcripts were amplified from serial dilutions of cDNA (1∶1, 1∶5 and 1∶25) in 35 cycles of PCR using primers previously described [20]. cDNA from the reaction without reverse transcriptase was used as a negative control for the PCR reaction. RNA derived from HE2 cells, treated with TPA to trigger virus reactivation, served as a positive control for viral gene expression. For PCR of viral gene expression from explant-derived tumor cells, viral gene transcripts were amplified from cDNA directly without dilution.

Germline immunoglobulin gene and postswitch transcripts were amplified by PCR as previously described [18]. For PCR of B cell-associated genes, the following primers were used to obtain the indicated products: AID-F: 5′-ACATCTCAGACTGGGACCTG-3′, AID-R: 5′-TCAAAATCCCAACATACGAAATG-3′; PAX5-F: 5′-AACTTGCCCATCAAGGTGTC-3′, PAX5-R: 5′-CTGATCTCCCAGGCAAACAT-3′; PU.1-F: 5′ CCCTCCATCGGATGACTTGGTTAC-3′, PU.1-R: 5′-GCTTCTCCATCAGACACCTCCAGG-3′; CD79a-F: 5′- GTGAAAACAATGGCAGGAA-3′, CD79a-R: 5′- AGGTTCAGGCCCTCATAGAG-3′; CD79b-F: 5′-TCTCAGAAGAGGGACGCATT-3′, CD79b-R: 5′- AATGTTCAAGCCCTCATAGG-3′; J chain-F: 5′-ATGAAGACCCACCTGCTT CTC-3′, J chain-R: 5′-GTCAGGGTAGCAAGAATCG GG-3′; GAPDH-F: 5′-CCATCACCATCTT CCAGGAG-3′, GAPDH-R: 5′-CCTGCTTCACCACC TTCTTG-3′.

### Reactivation stimulation and immunoblots

MHV68 immortalized FL cell lines were treated with 20 ng/ml 12-O-tetradecanoyl-phorbol-13-acetate (TPA), F(ab′)_2_ anti-mouse IgG (5 µg/ml), LPS (10 µg/ml), trichostatin A (5 µM), sodium butyrate (2 mM), or 5-azacytidine (5 µM) for 48 hr prior to the harvest. Immunoblot analyses were performed as previously described [20] to detect lytic viral antigen expression.

### Lymphoma induction in immunodeficient mice

The tumorigenic ability of MHV68 immortalized cell lines was tested in 6 to 8 week-old female Nu/J nude mice which were purchased from The Jackson Laboratory, or RAG2-/- mice which were bred at Emory University. Cells were suspended in PBS and injected subcutaneously or intraperitoneally at a concentration of 2.5-5×10^6^ cells in a volume of 0.2 ml. Control mice were injected with PBS. The animals were euthanized by carbon dioxide inhalation when visible tumors reached a diameter of 1-2 cm. All animals were autopsied and examined for metastases. Tumors were excised under sterile conditions and divided into two fragments, one of which was fixed in 10% neutral buffered formalin (Sigma) and processed for histopathology and immunohistochemistry and the other fragment was explanted into cell culture in RPMI 1640 medium with 10% FCS, 100 U/ml penicillin and100 µg/ml streptomycin.

## Supporting Information

Figure S1 **Presence of secreted IgG2a in the supernatants of MHV68 immortalized fetal liver-derived B cell lines.** Immunoglobulin isotyping of supernatants recovered from MHV68 immortalized B cell lines is shown. Supernatants were collected from different MHV68 immortalized cell lines at day 5 after passage as described in Materials and Methods.
(TIF)

Figure S2 **Presence of cytoplasmic IgG2a in MHV68 immortalized fetal liver-derived B cell lines.** MHV68 immortalized fetal liver cells were fixed and stained with anti-IgG1, -IgG2a, or -IgG3. DAPI was used to counterstain nuclei. Splenocytes from C57BL/6 mice treated as indicated were used as positive controls.
(TIF)

Figure S3 **Detection of both germline and post-switch immunoglobulin transcripts.** (A) Detection of germline and post-switch transcripts arising from the immunoglobulin heavy chain locus. A schematic illustration of germline and post-switch transcripts is shown. RT-PCR of germline transcripts containing I promoter and C_H_ exon of the same isotype are shown in the upper panel, while RT-PCR of IgG2a post-switch transcripts containing Iµ promoter sequences spliced to the C_2a_ exon is shown in the lower panel. Splenocytes from C57BL/6 mice treated as indicated served positive controls. (B) Nucleotide sequence of IgG2a post-switch transcript cloned from representative MHV68 transformed SL-1 cell line.
(TIF)

Figure S4 **Amino acid sequence alignment of Ig heavy chain variable region sequences amplified from two MHV68 transformed B cell lines.** Sequences from the SL-1 (denoted with the prefix H1) and SL-6 (denoted with the prefix H6) cell lines are shown. Sequences were aligned to the most closely related germ line V segment sequence. These analyses reveal the presence of multiple distinct variable region sequences in each cell line, demonstrating that these cell lines are not clonal.
(TIF)

Figure S5 **Detection of B cells in spleen and lymph node sections.** Sections from spleen, lymph node and subcutaneous (SC) tumors homogeneously express the B cell surface marker B220. Staining of tumors obtained from athymic nude mice are shown. Note, there is a low level of CD8+ T cells in mock treated nude mice in the spleen and lymph nodes, but these are not observed in the tumor sections. DAPI was used to counterstain nuclei.
(TIF)

Figure S6 **Explanted tumor cells exhibited similar viral gene and cell surface marker expression as parental cell lines.** (A) RT-PCR of viral gene expression for explant cells from subcutaneous tumors (SCT) and lymph node tumors (LNT). Two cell lines along with the cells explanted from two individual mice tumors derived from SC or IP injection are shown. (B) Explanted tumor cell lines express surface B220, IgG2a, MHC class II, CD5 and CD138.
(TIF)

Table S1 **Analysis of rearranged heavy chain VDJ junctions.** VDJ junctions were PCR amplified from cDNA prepared from 2 representative MHV68 immortalized B cell lines, as described in Materials and Methods.
(TIF)

Table S2 **Identification of germline segments rearranged in immunoglobulin heavy chain sequences.** From the analyses of rearranged heavy chain VDJ junctions, germline V and J segments were identified. In addition, in some cases the rearranged D segment could also be identified. In almost all cases the V segment sequences closely matched the germline V sequence.
(TIF)
