# Supplementary figures and images for: Murine Gamma-herpesvirus Immortalization of Fetal Liver-Derived B Cells Requires both the Viral Cyclin D Homolog and Latency-Associated Nuclear Antigen
Source: PLoS Pathog. 2011 Sep 8;7(9):e1002220. doi: 10.1371/journal.ppat.1002220 (PMC3169539; doi:10.1371/journal.ppat.1002220)

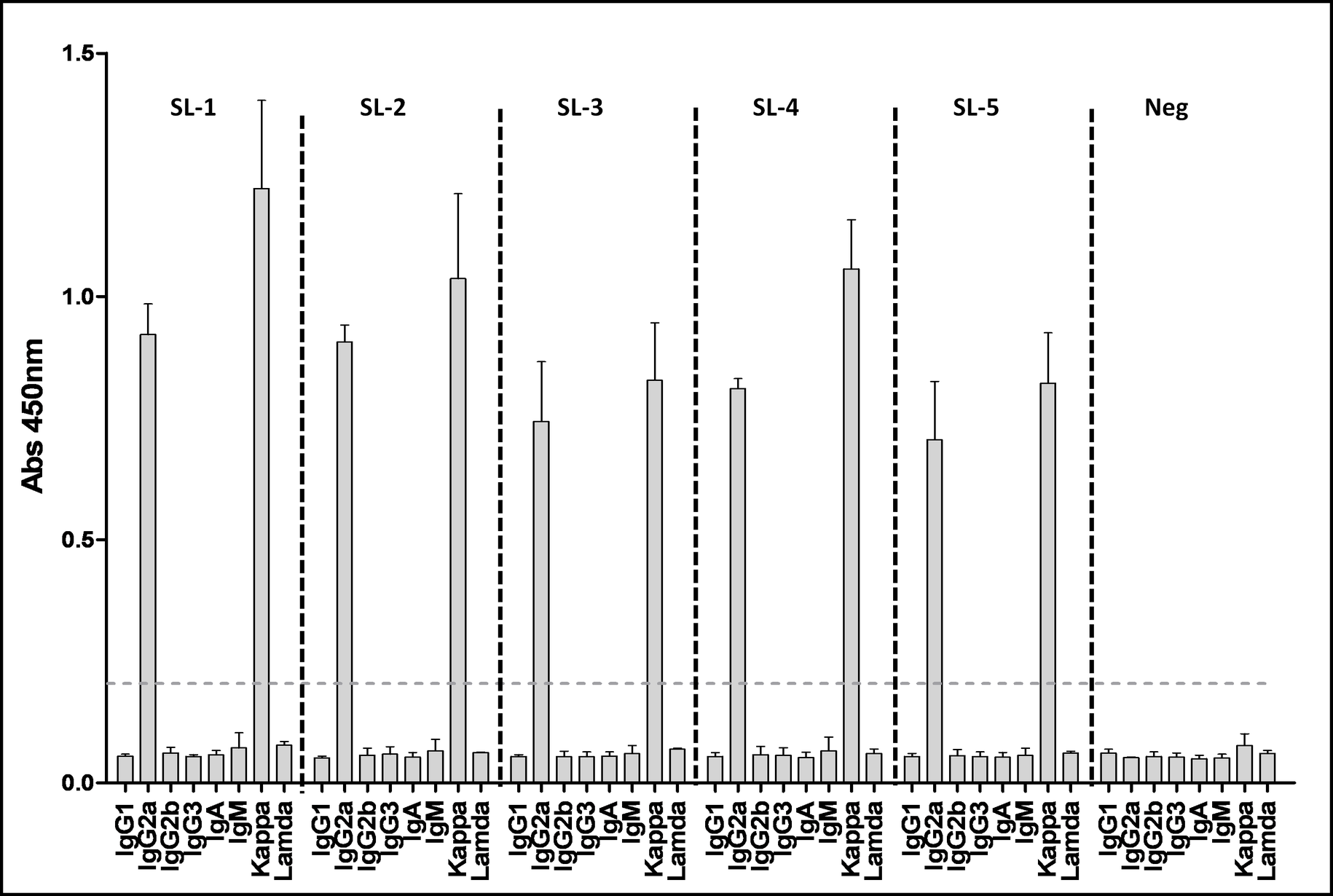

Supplement: Figure S1 — Presence of secreted IgG2a in the supernatants of MHV68 immortalized fetal liver-derived B cell lines. Immunoglobulin isotyping of supernatants recovered from MHV68 immortalized B cell lines is shown. Supernatants were collected from different MHV68 immortalized cell lines at day 5 after passage as described in Materials and Methods. (TIF) [file ppat.1002220.s001.tif]

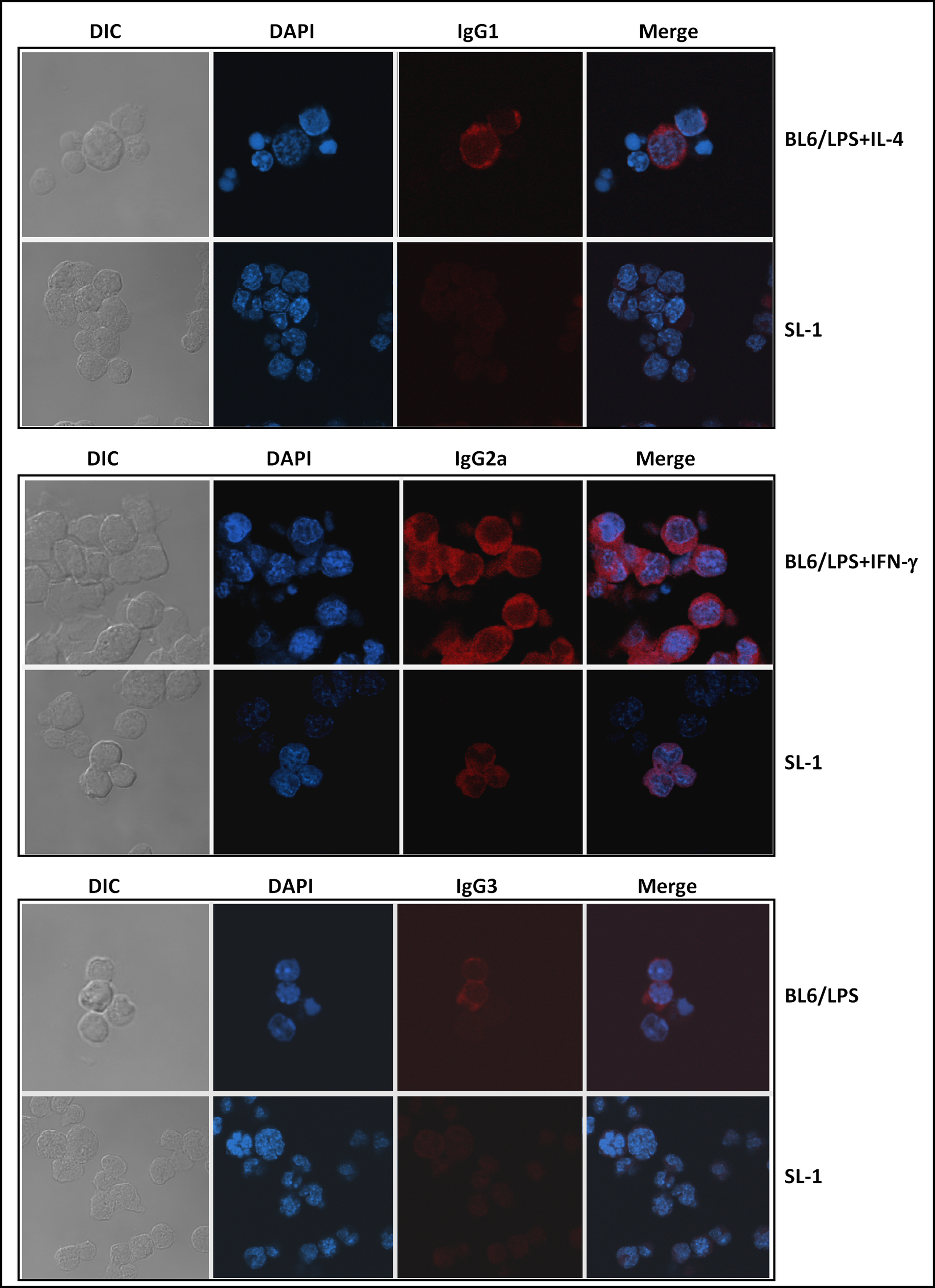

Supplement: Figure S2 — Presence of cytoplasmic IgG2a in MHV68 immortalized fetal liver-derived B cell lines. MHV68 immortalized fetal liver cells were fixed and stained with anti-IgG1, -IgG2a, or -IgG3. DAPI was used to counterstain nuclei. Splenocytes from C57BL/6 mice treated as indicated were used as positive controls. (TIF) [file ppat.1002220.s002.tif]

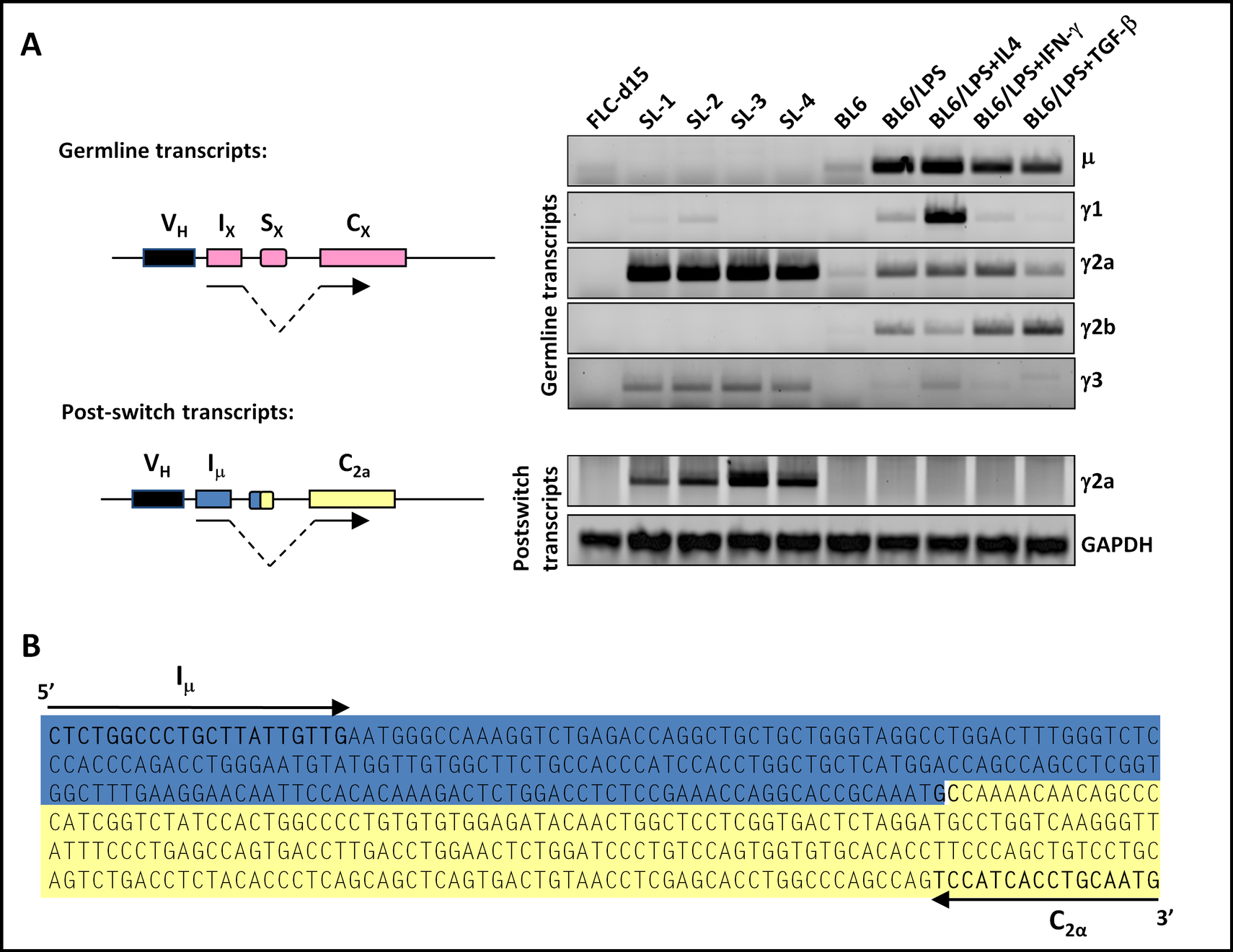

Supplement: Figure S3 — Detection of both germline and post-switch immunoglobulin transcripts. (A) Detection of germline and post-switch transcripts arising from the immunoglobulin heavy chain locus. A schematic illustration of germline and post-switch transcripts is shown. RT-PCR of germline transcripts containing I promoter and CH exon of the same isotype are shown in the upper panel, while RT-PCR of IgG2a post-switch transcripts containing Iµ promoter sequences spliced to the C2a exon is shown in the lower panel. Splenocytes from C57BL/6 mice treated as indicated served positive controls. (B) Nucleotide sequence of IgG2a post-switch transcript cloned from representative MHV68 transformed SL-1 cell line. (TIF) [file ppat.1002220.s003.tif]

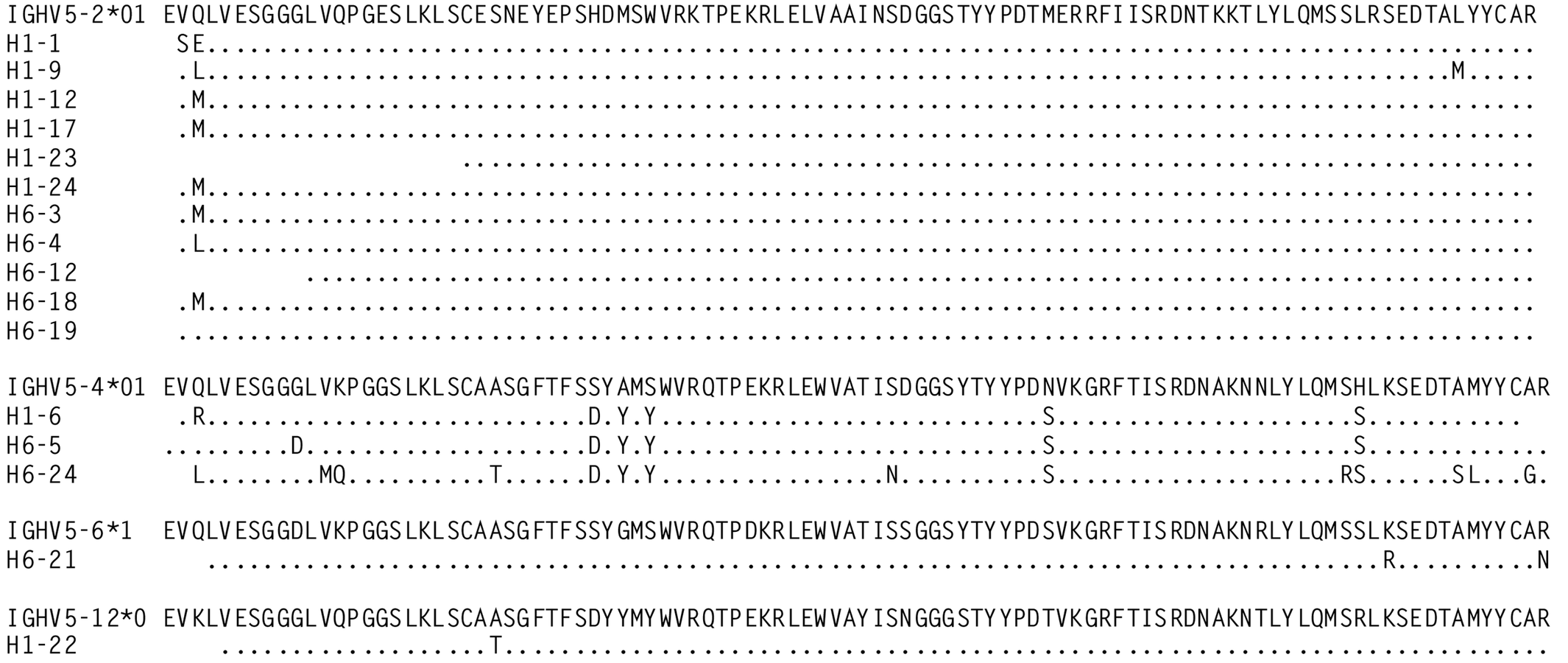

Supplement: Figure S4 — Amino acid sequence alignment of Ig heavy chain variable region sequences amplified from two MHV68 transformed B cell lines. Sequences from the SL-1 (denoted with the prefix H1) and SL-6 (denoted with the prefix H6) cell lines are shown. Sequences were aligned to the most closely related germ line V segment sequence. These analyses reveal the presence of multiple distinct variable region sequences in each cell line, demonstrating that these cell lines are not clonal. (TIF) [file ppat.1002220.s004.tif]

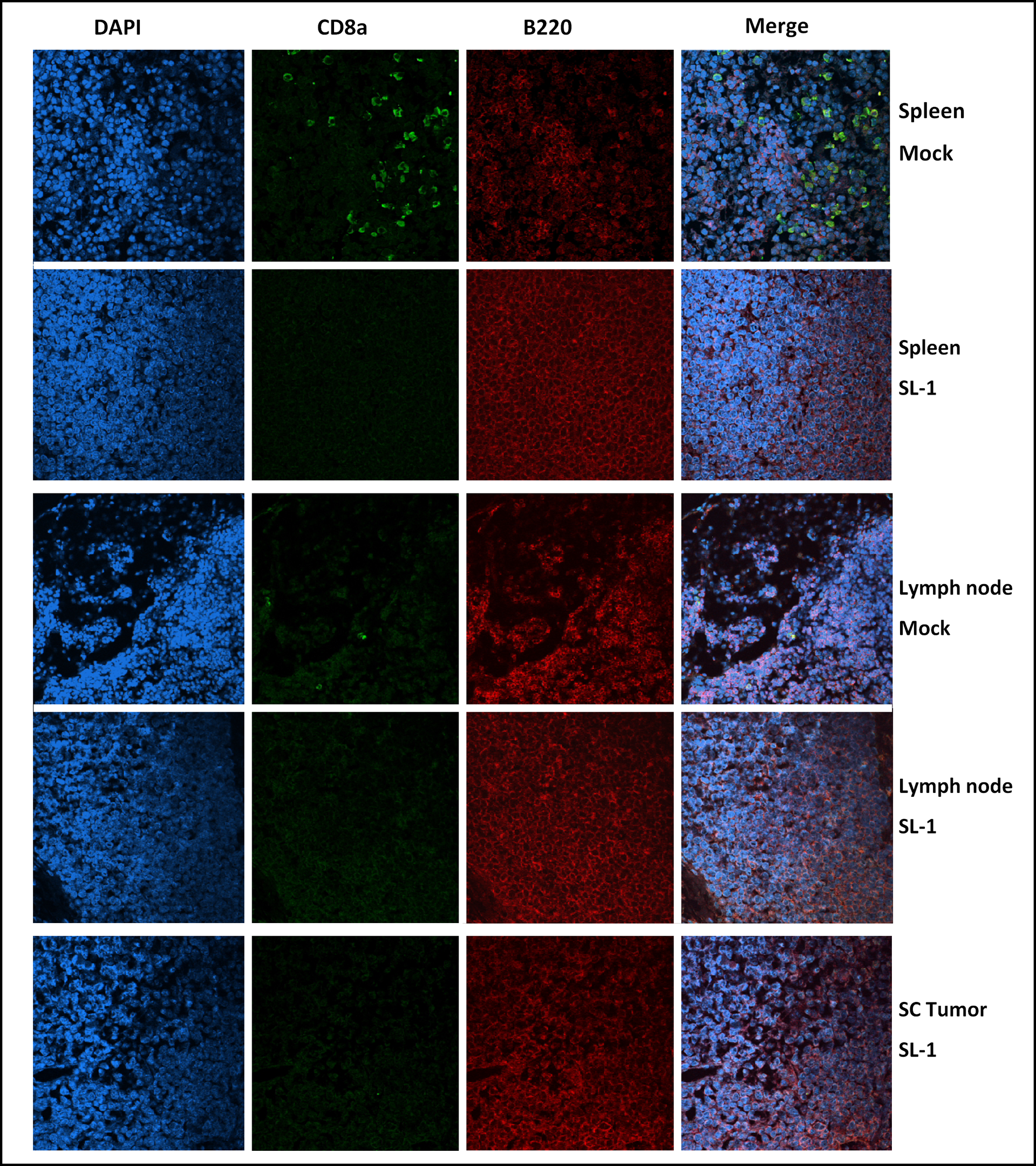

Supplement: Figure S5 — Detection of B cells in spleen and lymph node sections. Sections from spleen, lymph node and subcutaneous (SC) tumors homogeneously express the B cell surface marker B220. Staining of tumors obtained from athymic nude mice are shown. Note, there is a low level of CD8+ T cells in mock treated nude mice in the spleen and lymph nodes, but these are not observed in the tumor sections. DAPI was used to counterstain nuclei. (TIF) [file ppat.1002220.s005.tif]

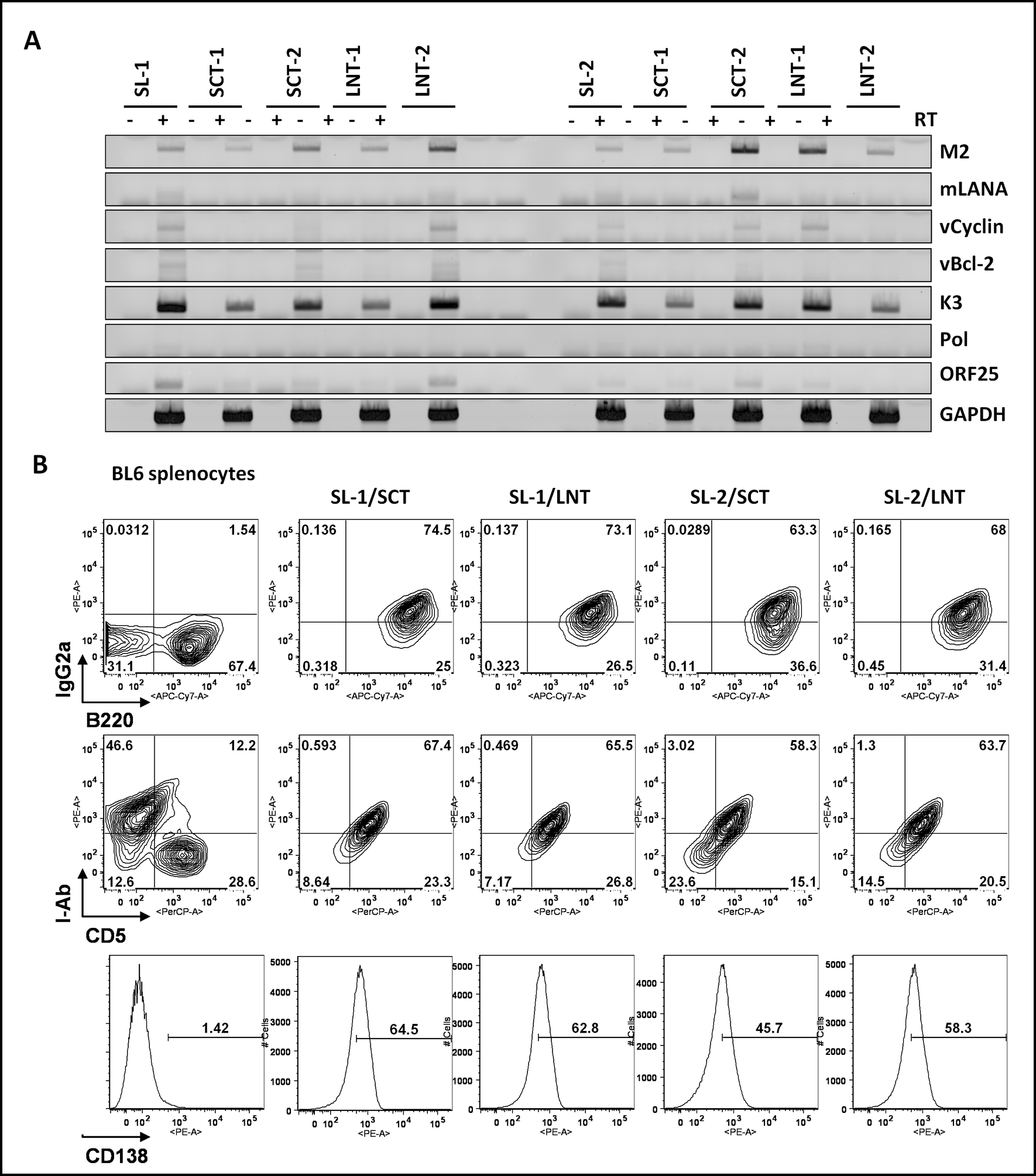

Supplement: Figure S6 — Explanted tumor cells exhibited similar viral gene and cell surface marker expression as parental cell lines. (A) RT-PCR of viral gene expression for explant cells from subcutaneous tumors (SCT) and lymph node tumors (LNT). Two cell lines along with the cells explanted from two individual mice tumors derived from SC or IP injection are shown. (B) Explanted tumor cell lines express surface B220, IgG2a, MHC class II, CD5 and CD138. (TIF) [file ppat.1002220.s006.tif]

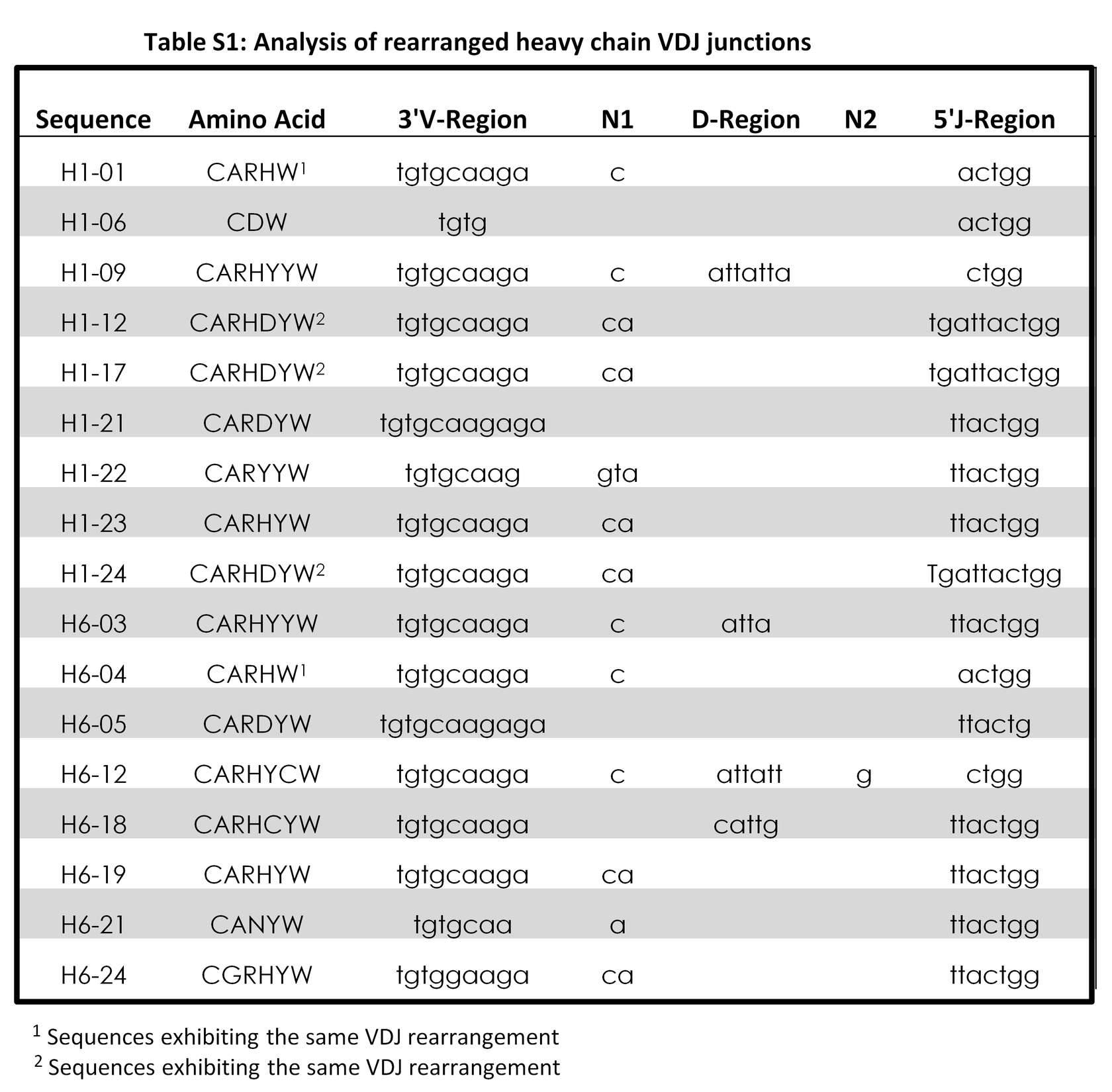

Supplement: Table S1 — Analysis of rearranged heavy chain VDJ junctions. VDJ junctions were PCR amplified from cDNA prepared from 2 representative MHV68 immortalized B cell lines, as described in Materials and Methods. (TIF) [file ppat.1002220.s007.tif]

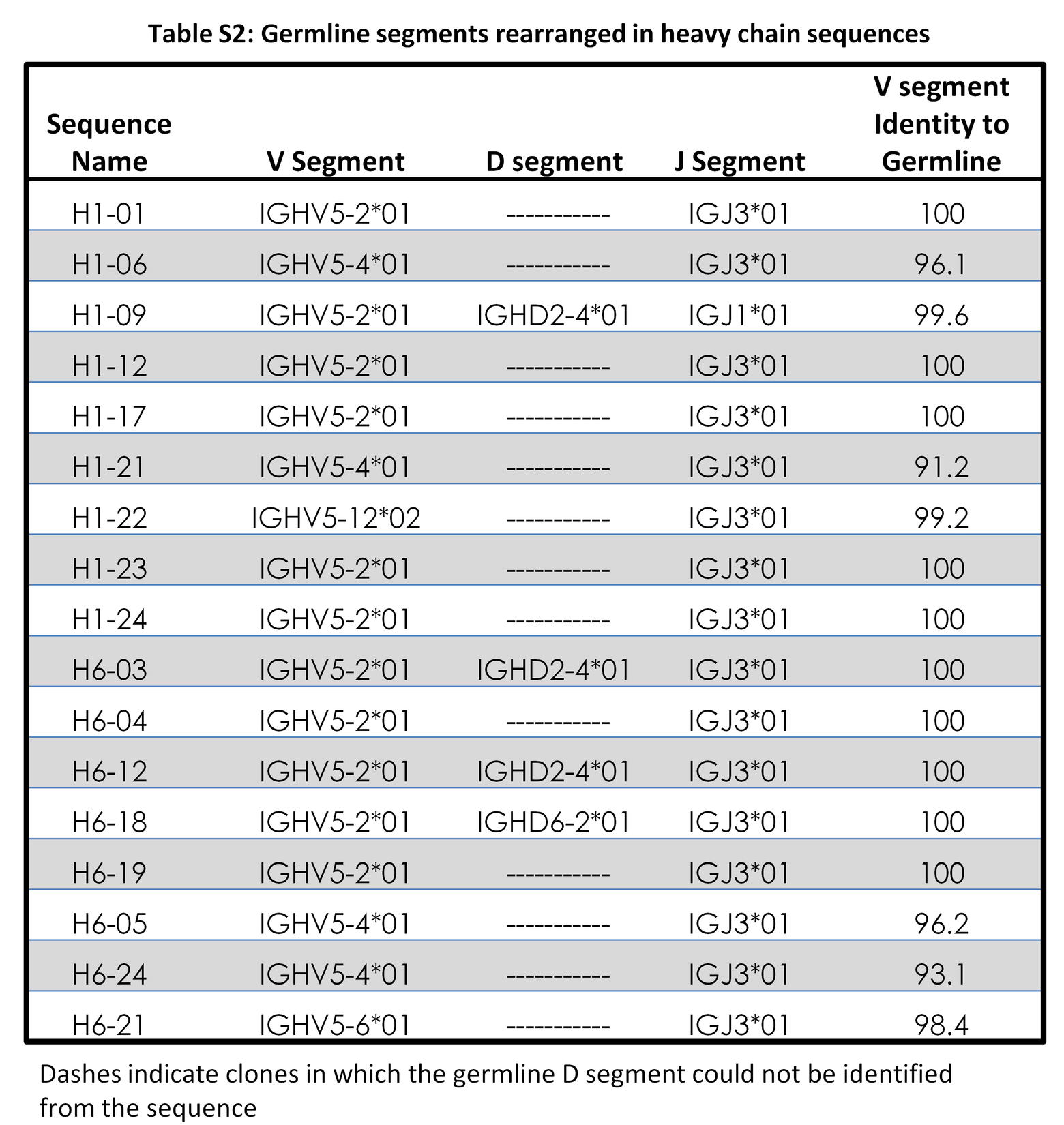

Supplement: Table S2 — Identification of germline segments rearranged in immunoglobulin heavy chain sequences. From the analyses of rearranged heavy chain VDJ junctions, germline V and J segments were identified. In addition, in some cases the rearranged D segment could also be identified. In almost all cases the V segment sequences closely matched the germline V sequence. (TIF) [file ppat.1002220.s008.tif]
